# The Genome Assembly and Annotation of the Apollo Butterfly *Parnassius apollo*, a Flagship Species for Conservation Biology

**DOI:** 10.1093/gbe/evab122

**Published:** 2021-06-11

**Authors:** Lars Podsiadlowski, Kalle Tunström, Marianne Espeland, Christopher W Wheat

**Affiliations:** 1 Centre for Molecular Biodiversity Research, Zoological Research Museum Alexander Koenig (ZFMK), Bonn, Germany; 2 Department of Zoology, Stockholm University, Sweden; 3 Centre of Taxonomy and Evolutionary Research, Zoological Research Museum Alexander Koenig (ZFMK), Bonn, Germany

**Keywords:** genome, ONT sequencing, Parnassius, conservation genomics, genome expansion

## Abstract

Conservation genomics has made dramatic improvements over the past decade, leveraging the power of genomes to infer diverse parameters central to conservation management questions. However, much of this effort has focused upon vertebrate species, despite insects providing similar flagship status with the added benefit of smaller genomes, shorter generation times and extensive historical collections in museums. Here we present the genome of the Apollo butterfly (*Parnassius apollo*, Papilionidae), an iconic endangered butterfly, which like many species in this genus, needs conservation genomic attention yet lacks a genome. Using 68.7 Gb of long-read data (N50 = 15.2 kb) we assembled a 1.4 Gb genome for the Apollo butterfly, making this the largest sequenced Lepidopteran genome to date. The assembly was highly contiguous (N50 = 7.1 Mb) and complete (97% of Lepidopteran BUSCOs were single-copy and complete) and consisted of 1,707 contigs. Using RNAseq data and Arthropoda proteins, we annotated 28.3K genes. Alignment with the closest-related chromosome-level assembly, *Papilio bianor*, reveals a highly conserved chromosomal organization, albeit genome size is highly expanded in the Apollo butterfly, due primarily to a dramatic increase in repetitive element content. Using this alignment for superscaffolding places the *P. apollo* genome in to 31 chromosomal scaffolds, and together with our functional annotation, provides an essential resource for advancing conservation genomics in a flagship species for insect conservation.

Significance

Species conservation is most successful when a high natural variation in the species is maintained. Genomic information of high quality is a prerequisite for modern population genetics and conservation genomics projects. Here we report the genome of the Eurasian Apollo butterfly *Parnassius apollo*, a species which saw a strong decline in many European countries during the 20th century. This will provide useful information for future conservation genomics studies.

## Introduction

The use of genomic scale data to inform upon conservation issues has dramatically increased over the past decade, giving rise to the fast-growing field of conservation genomics (Primmer 2009; Allendorf et al. 2010; Ouborg et al. 2010; Steiner et al. 2013; Benestan et al. 2016; Supple and Shapiro 2018; Hohenlohe et al. 2021). Aided by high-quality reference genomes, research projects now routinely use individual-level resequencing data to gain detailed insights into population structure, gene flow, inbreeding, genetic load, as well as admixture dynamics with closely related species (Hu et al. 2020; Wright et al. 2020). DNA from historical and ancient samples is providing insights into historical levels of genetic diversity, inbreeding and introgression, enabling important benchmarks for assessing species statuses today (Bi et al. 2013; van der Valk et al. 2019; Gauthier et al. 2020; Wu et al. 2020). Unfortunately, the vast majority of conservation genomic projects have been conducted with vertebrate systems, which have been the primary focus of many conservation management programs (Saremi et al. 2019; Eldridge et al. 2020). Insects, and butterflies in particular, present a unique opportunity given their detailed study, extensive historical samples from collections, generally smaller genomes, and much shorter generation times.

Much is known about the ecology, phylogenetic relationships, and biogeography of Apollo butterflies (Genus *Parnassius*, Papilionidae, Lepidoptera) (Nakonieczny et al. 2007; Condamine 2018; Condamine et al. 2018), but currently a genomic data set is lacking. Comprising about 40 species, the genus has a northern circumpolar and mainly montane distribution. The Mountain Apollo (*Parnassius apollo*) has a wide distribution mainly in mountain regions of the Palearctic—from Spain to Western China and from Norway to southern Italy and the Caucasus. More than 200 subspecies have been described, largely based on subtle differences in wing coloration (Glassl 2017). The 20th century saw a substantial decline of this species throughout Europe, mainly due to habitat loss in the heavily industrialized countries (Nakonieczny et al. 2007).

Today, the Mountain Apollo is the only nontropical butterfly on the CITES list (appendix II, https://cites.org/eng/app/appendices.php). Being a charismatic and easily recognizable species, it has become a special focus for conservation efforts in multiple countries, for example, France, Germany, Poland, Sweden (Nakonieczny et al. 2007). Currently, conservation managers need information regarding the inbreeding status of remnant populations and insights into which among these are suitable for restocking efforts, which are questions genomic tools can provide cost effective insights.

Although many butterfly species have a moderate genome size of 200 − 500 Mb, *Parnassius* species seem to be an exception in having much larger genomes; the estimated genome size for *Parnassius orleans* is 1.25 Gb (Liu et al. 2020). The closest relatives with genome data available are the swallowtails (*Papilio* sp.), which share a last common ancestor with *Parnassius* more than 50 Ma (Espeland et al. 2018; Allio et al. 2020), and with assembly sizes ranging between 230 and 400 Mb. Among these *Papilio* assemblies, the smaller assemblies relied solely upon Illumina short reads that likely underestimate repeat content and genome size, whereas the 400 Mb genome of *Papilio bianor* was assembled to high accuracy at the chromosome level using PacBio long reads and Hi-C data (Lu et al. 2019). Thus, there is at least a 3-fold increase in genome size in *Parnassius* compared with *Papilio*. This is a burden for genome sequencing cost and complicates the assembly and annotation process, but also provides more sites with variation to inform population genomics analyses.

Here we present the first genome assembly of *P. apollo*, generated with a long-read sequencing approach, complemented with genomic polishing using short-reads and RNAseq data to facilitate annotation. Analyses of repeat content and synteny in comparison with a high-quality swallowtail genome sheds light on the genome expansion process in Apollo butterflies. This reference genome will enable future population genomics studies with Apollo butterflies.

## Results and Discussion

### Genome Sequence Statistics

**Fig. 1:**
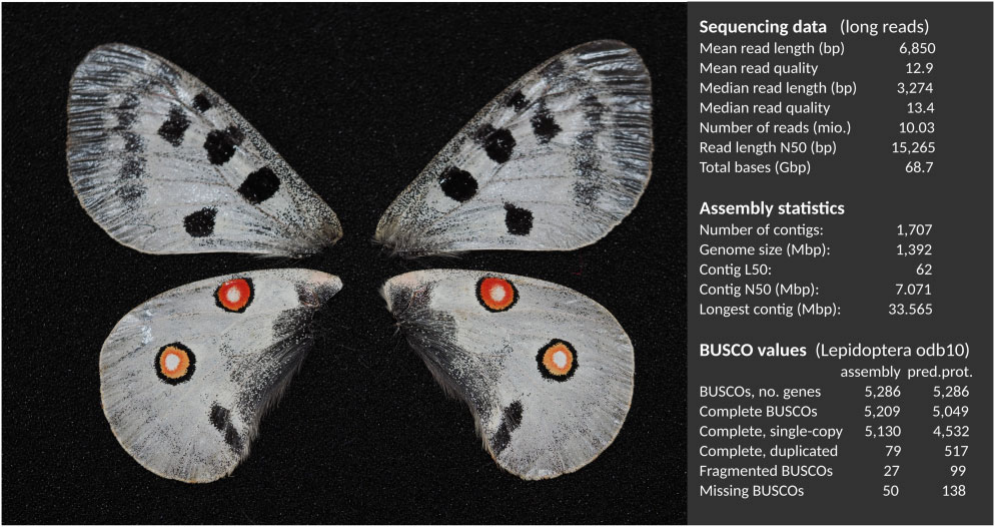
Wing voucher of the specimen of *P. apollo* used for long-read sequencing; sequence data, assembly and BUSCO statistics.

Long-read sequencing (Oxford Nanopore) yielded an output of 10 million reads (68.7 Gb, N50 = 15.2 kb) for the Apollo butterfly (fig. 1), for an estimated genome coverage of 49.1×, assuming a genome size of 1.4 Gb for the Apollo butterfly. We generated 98.6 million reads of Illumina data from genomic DNA (14.7 Gb), a coverage of roughly 10× as assumed from assembly size. Illumina data were used to correct remaining nonrandom sequencing errors frequently associated with ONT long-read data. The resulting polished genome consisted of 1.39 Gb spread across 1,707 contigs with an N50 of 7.1 Mb and BUSCO score of >98%, with 97% of genes being single-copy and complete (fig. 1). A superscaffolded assembly using the chromosome-scale assembly of *P. bianor* as reference resulted in a slightly lower number of contigs (1,451) and massively increased the N50 to 40.9 Mb (see below for details on the high synteny between these species, which justifies superscaffolding). Although being the largest Lepidopteran genome sequenced to date, our estimated genome size was close to that estimated from flow cytometry for a related *Parnassius* species (Liu et al. 2020).

Illumina reads from RNAseq experiments (male, female, larva) sum up to 75 million reads (11.2 Gb). An average of 99.12% of the three libraries of RNAseq mapped to the genome, which we used alongside Arthropoda proteins for annotation model training. Protein prediction yielded 28,334 genes and 30,102 transcripts. BUSCO analysis of the annotation resulted in 95.5% complete BUSCOs (single copy: 85.7%, duplicated: 9.8%).

### Comparative Analysis

**Fig. 2:**
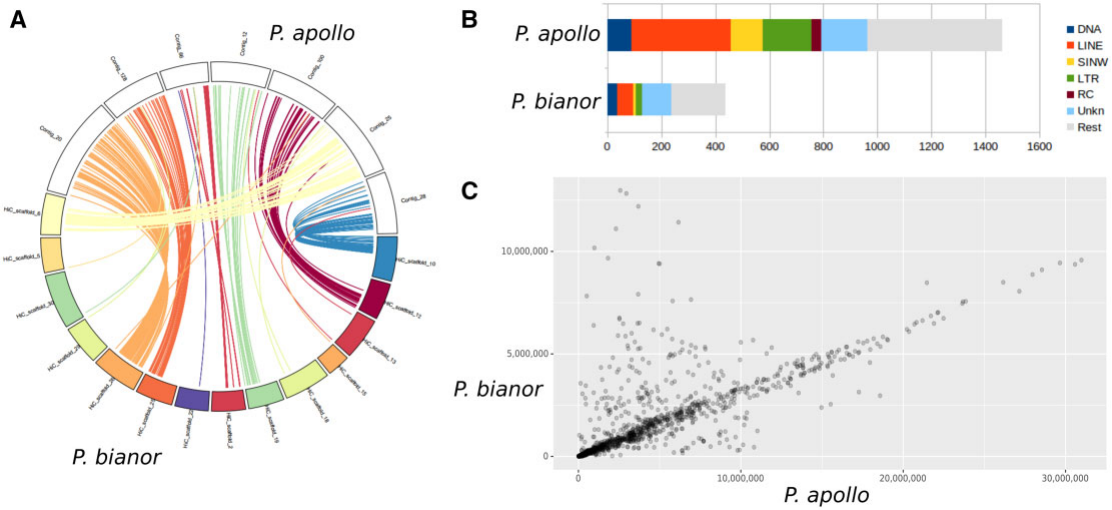
Comparison of the assemblies of *P. apollo* and *Papilio bianor*. (*A*) Synteny between selected chromosomes and contigs. (*B*) Repeat content of the two assemblies (DNA, DNA transposons; RC, rolling circle elements; UnKn, unknown repeat elements). (*C*) Distances (bp) of homologous BUSCO gene pairs found on the same chromosome or contig in both assemblies.

To assess the genome size expansion in *P. apollo*, we compared this genome against that of *Papilio bianor* (Lu et al. 2019), the closest relative with a high-quality, chromosome-scale assembly. Whole-genome alignment revealed a high degree of chromosomal synteny between the species, suggesting no large-scale chromosomal rearrangements between these species, consistent with the vast majority of Lepidoptera (Ahola et al. 2014; Hill et al. 2019). Given this high degree of synteny, this alignment allowed us to place 79.9% (1.11 Gb) of the *P. apollo* genome into a chromosomal framework. On average, we find that the *P. apollo* chromosomes are three times larger than their *P. bianor* counterparts (fig. 2*A*).

We next estimated the repeat content of the *P. apollo* genome (65.2%) and compared this with *P. bianor* (55.3%), finding that it had many of the usual suspects (fig. 2*B*). However, although several of the repeat classes that expanded were also those that were the largest in *P. bianor*, other classes went from being relatively rare in *P. bianor* to common in *P. apollo* (e.g., SINEs and rolling-circle transposons). There is a disproportionately higher amount of LINEs and LTRs in *P. apollo* than expected from genome expansion alone.

We then conducted a more detailed analysis of the shared single-copy orthologs (from the BUSCO Lepidoptera set) between these two species by estimating the chromosomal distances between flanking genes on the same chromosome or contig (*n* = 1,142; fig. 2*C*). There is a clear correlation of distances between gene pairs on the same contig or chromosome between the two species, with an almost threefold higher distance in *P. apollo*. This corresponds roughly with the genome size difference, with *P. apollo* having a genome of about 3.3-fold size compared with *P. bianor*.

## Conclusions

Here we present a high-quality genome assembly for the Apollo butterfly, *Parnassius apollo*, an iconic, rare, and endangered species. With 1.39 Gb, it is the largest Lepidopteran genome published so far. Comparative assessment and assembly metrics indicate a highly contiguous and accurate assembly for which we generated a functional annotation. The genome expansion is associated with an increase in repeat elements at frequencies consistent with related species. This genome will serve as an important resource to the numerous ongoing conservation efforts for *P. apollo*, and its congeners, including many endangered species around the world.

## Materials and Methods

### DNA Extraction and Sequencing

A male *P. apollo* sample was collected in northern Italy, in the village of Etirol (Valle d'Aosta, Comune di Torgnon). Samples were placed in ethanol (70%) upon collection and stored at -20 °C until laboratory analysis. For extracting high molecular weight (HMW) DNA we used half of the whole thorax as input for the Nanobind Tissue Big extraction kit (Circulomics, MD). The tissue was first washed and rehydrated in an ethanol removal buffer as recommended by Circulomics. The rehydrated tissue was then submerged in liquid nitrogen and ground with a ceramic pestle until it turned into fine dust, followed by the Circulomics extraction protocol instructions. The resulting HMW DNA was treated with the Short Read Eliminator Kit XS (Circulomics) to reduce sequences below 10 kb long. Final DNA purity and concentrations were measured using Nanodrop (ThermoFisher, MA) and Qubit (ThermoFisher).

Sequencing libraries were constructed using the HMW DNA as input for the Nanopore LSK-110 ligation kit (Oxford Nanopore Technologies, UK) following the manufacturer’s protocol with the following modifications suggested by Circulomics: NEB end-prep and repair times were extended 6× to 30 min at 20 °C and 30 min at 65 °C, adapter ligation time was extended to 1 h, and the elution of magnetic beads was extended to 20–60 min, depending on the sample. All DNA extractions and sequencing library preparations were carried out in the laboratories at the Department of Zoology of Stockholm University. We used a total of four new MinION R9.4.1 flow cells and one partially used R9.4.1 flow cell, with 1 nuclease wash for each run (except for the partially used R9.4.1 flow cell). All sequencing was performed in the modern laboratory facilities at the Centre for Palaeogenetics, Stockholm University where DNA cross-contamination is minimal.

### De Novo Assembly

Raw ONT sequence data were first basecalled using Guppy v4.2.2 (community.nanoporetech.com), then assembled using the Shasta long-read assembler (Shafin et al. 2020; v0.7, modified version of NanoporeSep2021 config). We expected a genome size of >1 Gb for the Apollo butterfly, based upon estimates of 1.25 Gb from flow cytometric determination methods with another species of the genus, *P. orleans* (Liu et al. 2020). The resulting draft assemblies were then polished with the same ONT sequence data used for the assembly with the pepper-polish pipeline v0.1 (github.com/kishwarshafin/pepper), to improve base accuracy and reduce assembly errors. The assembly was additionally polished with Illumina short-read data generated from an ethanol-preserved male collected in Germany with POLCA (from MaSuRCA v.4.0.2) (Zimin and Salzberg 2020). Finally, the polished draft assembly was filtered for alternative haplotypes using purge_dups v.1.2.5 (Liu et al. 2020), resulting in a haploid genome assembly. Superscaffolding was performed using Ragtag (github.com/malonge/RagTag; a successor of RaGoo; Alonge et al. 2019) with the genome of *Papilio bianor* as reference genome (Lu et al. 2019; downloaded from GigaScience repository).

### Quality Assessment

We assessed assembly quality using QUAST (Gurevich et al. 2013), the stats.sh utility in bbmap v.38.08 (Bushnell B, sourceforge.net/projects/bbmap) and BUSCO v.3.0.2 (Simão et al. 2015) with the “lepidoptera_odb10” data set for the Apollo butterfly. Synteny comparison with a published assembly of *Papilio bianor* (Lu et al. 2019), was done using the *nucmer* utility included in MUMmer4 (Marcais et al. 2018), as this species is the most closely related to our focal species with a chromosomal level genome assembly. Candidates for contaminations were checked using short-read coverage of contigs and BLAST comparisons with Uniprot (UniProt Consortium 2021), using the Blobtoolkit (Challis et al. 2020).

### Genome Annotations and Repetitive Content

The final polished genomes were assessed for repetitive content using RepeatModeler and RepeatMasker (Flynn et al. 2020), and then annotated with BRAKER2 (Brůna et al. 2021) automated annotation pipeline using RNAseq data from larvae, adult males and adult females of *P. apollo*, as well as the Arthropoda protein data set from OrthoDB v.10 (Kriventseva et al. 2019) to train the algorithm. Functional annotation was done using EggNog mapper v.2.0.8-2 (Huerta-Cepas et al. 2017) against the eggNOG database v.5.0.1 (Huerta-Cepas et al. 2019) using Diamond v.2.0.6 (Buchfink et al. 2015).

### RNA Isolation and Sequencing

Tissue of adults (male, female) and larvae (sex not determined) from lab reared *P. apollo* samples from the Moselle valley, Germany, were initially stored in RNA later. RNA extraction was performed with a spin column method using Qiagen RNeasy mini kits (Qiagen, Hilden, Germany) following the manufacturer’s protocol. Total RNA was sent to a sequencing company (STARseq, Mainz, Germany), which performed the purification of mRNA, library preparation, and subsequent short read sequencing (Illumina Nextseq platform).

## Data Availability

The final genome assembly and annotation have been archived on ENA under the project number PRJEB44393. Also available on ENA are the Oxford Nanopore Technology MinION fastq sequences used for the assembly (accession number ERS6264556). Illumina raw reads are deposited at NCBI sequence read archive; the genomic reads used for polishing of the assembly are stored with accession number SRR6679361, transcriptome raw reads used for the annotation are archived under the project number PRJNA723476, with accession numbers SRR14292667-69.
